# Psychometric properties of instruments assessing exercise in patients with eating disorders: a systematic review

**DOI:** 10.1186/s40337-020-00315-2

**Published:** 2020-09-02

**Authors:** Astrid Harris, Phillipa Hay, Stephen Touyz

**Affiliations:** 1 School of Psychology, University of Sydney, Sydney, Australia; 2 Translational Health Research Institute, School of Medicine, Western Sydney University, Penrith, Australia; 3 InsideOut Institute and School of Psychology, University of Sydney, Sydney, Australia

**Keywords:** Eating disorders, Exercise, Factor analysis, Psychometrics, Validity

## Abstract

**Background:**

Research has identified factors specific to exercise in eating disorder patients such as affect regulation and compulsivity. Existing measures of exercise behaviour which were not originally designed for eating disorder patients may not adequately assess these factors. The aim of this systematic review is to identify and assess the psychometric properties of all self-report measures of exercise designed to be used with eating disorder patients.

**Method:**

A systematic review was conducted following the PRISMA guidelines. MedLine, Scopus and PsycINFO were systematically searched. A total of 12 studies examining two measures, the Exercise and Eating Disorders and the Compulsive Exercise Test, met inclusion criteria.

**Results:**

Validation studies showed promising results for both tests and established internal consistency, concurrent and convergent validity, and construct validity. The factor structure of the Compulsive Exercise Test was not confirmed in the majority of the studies included in this review, while there are only two studies conducting factor analysis on the Exercise and Eating Disorders.

**Conclusion:**

The two measures identified by this systematic review represent the current research on measures of compulsive exercise for eating disorder patients. Further research is needed to confirm a factor structure and validate both the Compulsive Exercise Test and the Exercise and Eating Disorders in more diverse clinical samples.

## Plain English summary

Exercise has long been recognised as an important feature of eating disorders. Research has consistently found that many people with eating disorders exercise because they feel a drive to exercise, or in order to regulate their emotions. This type of exercise, called ‘compulsive exercise’ can have a detrimental impact on peoples’ health and well-being. Compulsive exercise in eating disorders has been found to be associated with a range of adverse outcomes such as longer hospitalisation, higher risk of relapse, and higher risk of a chronic outcome. In order to treat exercise as a symptom of eating disorders, clinicians need a way to measure exercise behaviours specific to eating disorders. There are a number of tests that measure exercise behaviours, however most of them were not designed for the needs of eating disorder patients. The current review therefore examines the literature in order to identify and assess measurement tools for patients with eating disorders.

## Background

Exercise has long been described as an important feature of eating disorders [1, 2]. Despite its relevance, both terminology and aetiology remain unclear, and there is no consensus on what constitutes problematic exercise behaviours, and no widely accepted clinical criteria [3, 4]. A number of terms describing problematic exercise behaviour can be found both within and outside of an eating disorder context: ‘obligatory exercise’ [5, 6]**,** ‘compulsive exercise’ [4, 7, 8], ‘excessive exercise’ [9], ‘exercise addiction’ [10, 11] or ‘exercise dependence’ [12, 13]. While some of these terms refer to the quantitative (frequency, intensity, duration) component of exercise, others address the qualitative dimension (motivation, psychological experience) [4, 9]. Whether these terms capture the same construct remains unclear, and while they may overlap, there are clear distinctions between the various definitions [4]. There is evidence that the term ‘compulsive exercise’ may best describe exercise behaviour typically exhibited by eating disorder patients [14], hence this term is used throughout this paper unless when citing studies whose authors have explicitly used different terms. Research has found both anecdotal and empirical evidence supporting the term ‘compulsive exercise’ [4]. For example, eating disorder patients have described their exercise behaviour as ‘obsessive’, ‘driven’ and ‘out of control’ [15] and have indicated that they are unable to stop the behaviour even if they want to [16]. There are many operational definitions to be found in the literature, however the components they include appear to mostly overlap with the American Psychiatric Association’s conceptualisation of compulsivity, which is defined as ‘repetitive behaviours that the person feels driven to perform’ that are ‘aimed at preventing or reducing distress’ [17].

While a widely accepted working definition and maintenance model of compulsive exercise in eating disorders is still lacking, recent research has identified multiple factors playing a role in its development and maintenance. Exercise in eating disorders has long been thought to be a unidimensional construct with weight and shape concerns at its core [8, 16, 18], but more recent research has identified additional relevant factors such as compulsivity and affect regulation [19–21].

Multiple contemporary studies suggest that there is a link between high levels of exercise and compulsivity [2, 14, 19]. Compulsive exercise behaviour is characterized by an internal drive to exercise, rigid and inflexible exercise schedules, favouring exercise over other activities, and an inability to reduce or stop exercising despite possible negative outcomes [22–24]. Compulsive exercise has been shown to be associated with eating disorder features such as shape and weight concerns and drive for thinness [5, 14, 25]. Furthermore, high-level exercisers both with and without eating disorders score higher on measures of compulsivity than normal level exercisers [19, 26].

Exercising to avoid negative affect has consistently been shown to be a contributing factor to the maintenance of eating disorders [27, 28], and managing negative affect has been identified as one of the major reasons for continuing to exercise among eating disorder patients [29, 30]. There is also considerable evidence that exercise deprivation can lead to withdrawal symptoms [31–34]. Negative affective states when unable to exercise can include guilt, depression, irritability, restlessness, and anxiety [32, 34], and eating disorder patients may exercise to avoid experiencing negative emotions caused by exercise withdrawal [23].

Prevalence rates of increased physical activity in adults with eating disorders were found to range from 39 to 45.5% across eating disorder diagnoses and from 37 to 80% in restrictive type anorexia nervosa patients. Similar ranges can be found for other eating disorder subtypes [8, 35]. Among adolescent eating disorder patients, prevalence rates vary and may be as high as 85.3% [27]. While some studies have found differences in prevalence depending on eating disorder subtype [35], other studies have found no significant differences between eating disorder diagnoses [36]. This contradiction may be due to studies using a variety of different definitions and measures of physical activity [36]. For instance, the studies on prevalence rates used ‘compulsive exercise’ [8, 27], ‘excessive exercise’ [35] and ‘high-level exercise [36].

Compulsive exercise has consistently been found to be related to elevated eating psychopathology [5, 30], and in particular, to weight and shape concerns [36], dietary restraint [1]**,** drive for thinness [5] and body dissatisfaction [7]. Exercise in eating disorders is also associated with a variety of negative outcomes such as longer hospitalisation [36], higher risk of and earlier relapse [37, 38], higher risk of a chronic outcome [38], suicidality [39], and treatment drop-out [40]. Exercise can precede the onset of an eating disorder [16] and is often one of the last remaining symptoms [41].

The findings mentioned above clearly highlight the multidimensional nature of exercise in eating disorders. Given the high prevalence rates of compulsive exercise and associated negative outcomes, reliable psychometric instruments are a necessity when trying to identify and treat these behaviours [23]. However, data on compulsive exercise has been inconsistent due to the use of different instruments assessing exercise behaviours [14, 22], which all rely on different underlying definitions of the construct. Some examples include the Obligatory Exercise Questionnaire [42], a 20-item questionnaire which measures subjective need to exercise repetitively and assesses exercise frequency and intensity, feelings related to exercise and preoccupation with exercise [5]. The Compulsive Exercise Test, a 24-item questionnaire, was developed for use with eating disorder patients and addresses domains identified by research to play a role in exercise behaviours of these patients: compulsivity, affect regulation and shape and weight concerns [23]. The Commitment to Exercise Scale [9], an 8-item questionnaire, measures psychological commitment to exercise and addresses three components of exercising: negative affect when unable to exercise, exercising despite being unwell, and the degree to which exercise interferes with social commitments. The Exercise Addiction Inventory [11], a 6-item questionnaire developed for use as a screening tool, was designed to measure the degree of addiction to exercise by assessing for different components of addiction, including salience, mood modification, tolerance, withdrawal, conflict and relapse [10], while the Exercise Dependence Scale [12], a 29-item questionnaire, views exercise dependence as similar to substance dependence and is therefore based on the DSM-IV criteria of substance abuse. While these measures have been used with eating disorder patients and have been shown to distinguish between patients and controls [5, 22, 43, 44], it remains unclear whether they capture the idiosyncrasies specific to exercise in eating disorder populations [3], particularly in light of research identifying components such as affect regulation and compulsivity, which may not be reflected in the measures available. Hence this review was designed to identify measures specifically developed for assessing exercise in eating disorder patients as a subgroup of exercisers.

Focusing on quantitative rather than qualitative aspects when measuring exercise may be inadequate to capture the features specific to exercise in eating disorders [4], as quantitative aspects of exercise appear not to be related to eating psychopathology in both clinical and non-clinical samples [2, 14, 45]. These results indicate that frequency, intensity and duration of exercise may be less problematic than other motives such as a compulsive drive to exercise [2, 4, 14, 46]. In addition, researchers have been unable to agree on a quantitative threshold for problematic exercise behaviours [4]. Suggestions have included exercising at least five times a week for at least 1 h without stopping [47], exercising for more than 3 h on any given day [35], or exercising for at least 6 h a week [19]. It therefore appears that endeavouring to define problematic exercise behaviours in terms of quantitative factors may result in flawed definitions, particularly as not all quantitatively high amounts of exercise are compulsive, and not all compulsive exercisers show high frequency, intensity and duration of exercise [4].

It is therefore unlikely that the measures mentioned above all relate to the same underlying construct, and, with the exception of the Compulsive Exercise Test, none of them were designed specifically for use in an eating disorder context. The variety of definitions and measures has made it difficult to compare results across studies, and to judge whether results are relevant to eating disorder patients as a specific subgroup of exercisers [3]. Available measures may therefore not adequately capture problematic exercise behaviour as an eating disorder symptom. There is a clear need for measures of compulsive exercise that take into account eating disorder pathology and issues common to exercisers with eating disorders [23]. While a recent systematic review has highlighted the importance of affect regulation and compulsivity when studying exercise in eating disorder patients [20], no review has so far reported on available measures of compulsive exercise. The current review therefore aims at identifying and assessing all self-report measures of exercise designed to be used within an eating disorder context.

## Method

A systematic review was conducted following the PRISMA guidelines [48]. The search strategy was designed to find all studies which used a self-report measure of compulsive exercise developed to be used within an eating disorder context and assessed its psychometric properties. The inclusion criteria for studies used in this review were: (1) published in a peer reviewed journal (2) published in English, German or French, (3) studies used human subjects who either did not meet criteria for a clinical diagnosis or met criteria for an eating disorder as defined by DSM-5 and ICD-11 (4) studies used a self-report measure for compulsive exercise designed to be used within an eating disorder population and (5) studies assessed psychometric properties of these measures. No restrictions were based on demographic data such as sex, age, BMI, age of onset or duration of the eating disorder or whether participants had received treatment. No limitations were placed on publication year.

**Fig. 1:**
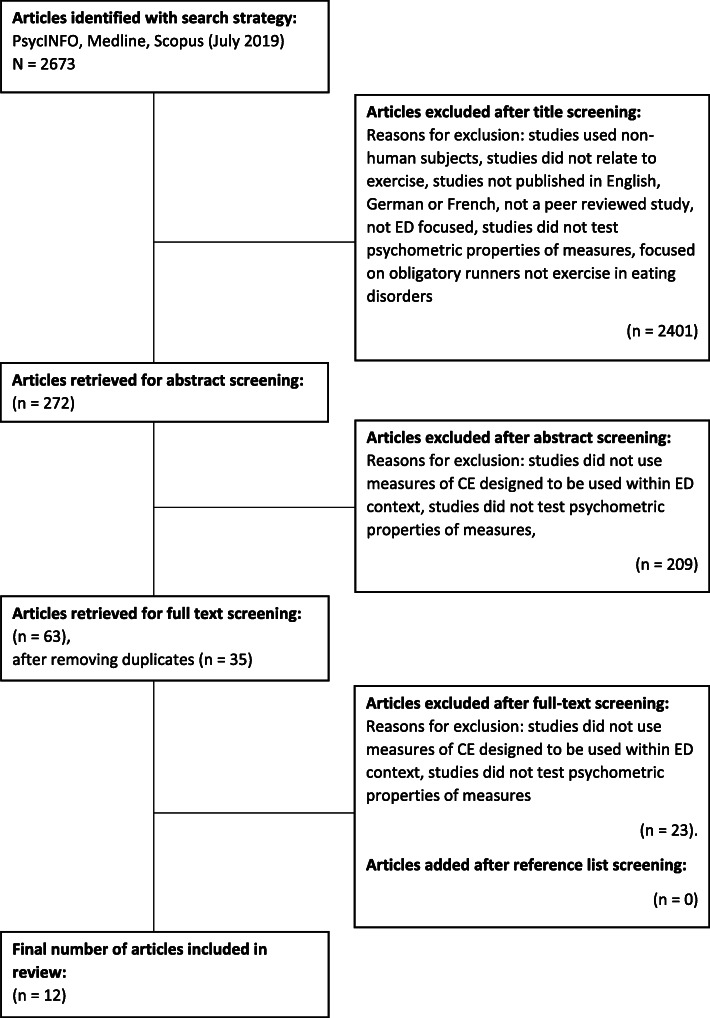
Flow diagram of article retrieval process

A search of published studies was conducted in July and August 2019 using the following electronic databases: PsycINFO (1806 – present), MedLine (1946 – present) and Scopus (1966 – present). Search terms were grouped into three categories (Group 1: Exercise addiction OR exercise dependence OR obligatory exercise OR compulsive exercise OR excessive exercise OR driven exercise OR maladaptive exercise OR pathological exercise OR obsessive exercise OR over-exercise OR exercise OR physical activity OR physical fitness OR running AND Group 2: psychometric OR self-report OR questionnaire OR measurement OR interview OR inventory OR assessment AND Group 3: eating disorders OR anorexi* OR bulimi* or binge eating OR EDNOS). Search terms were mapped to subject headings where possible. All subject headings were exploded. In addition, reference lists of studies selected for full-text screening were searched manually to identify any studies that were missed by the search strategy. The initial search yielded 2673 studies. Based on the title, 2401 studies were excluded, and 209 were excluded based on the abstract. A total of 63 articles were retained for full-text screening. After removing duplicates, a total of 35 articles remained. Twenty four articles did not meet the inclusion criteria, therefore a total of 12 articles were included in the review. For details see Fig. 1. A second reviewer screened a proportion of the titles and abstracts to minimise selection bias. Any disagreement was resolved by consensus.

Study quality was assessed using the Modified Quality Index [49]. Changes to the original version included removing items that were only relevant to intervention studies such as blinding or randomisation. As no intervention studies were included in this review, the Modified Quality Index was deemed appropriate to assess study quality. The Modified Quality Index includes 15 items which can be scored either 1 (*Yes*) or 0 (*No/ unable to determine*). Items 5 and 14 from the Modified Quality Index were removed, as they were deemed irrelevant to the studies incuded in this paper. Results of the study quality assessment can be found in Table 1. Studies were generally found to be of good quality with low risk of bias excepting power, with no study using power calculations to determine sampling adequacy. Only one study reported exact probability values. In order to minimise meta-bias, measures were also assessed on whether they were tested beyond the research group in which they were initially developed.

**Table 1:** Quality Index of included studies (Ferro & Speechley, 2009)

Quality Index Items														
Author (Year)	Hypotheses clearly described	Outcomes clearly described	Characteristics of patients described	Main findings clearly described	Actual probability values used	Response rate clearly described	Patients representative of population	Final sample representative of population	Staff, place and facilities representative	Evidence of data dredging	Statistical tests appropriate	Outcome measures valid/ reliable	Sample size or power calculation	Total Index Score
Danielsen (2012) [3]	1	1	1	1	0	1	1	1	0	1	1	1	0	10
Danielsen (2015) [50]	1	1	1	1	0	0	1	0	0	1	1	1	0	8
Danielsen (2018) [51]	1	1	1	1	0	1	0	0	0	1	1	1	0	8
Taranis (2011) [23]	1	1	1	1	0	0	0	0	0	1	1	1	0	7
Formby (2014) [52]	1	1	1	1	0	0	1	0	0	1	1	1	0	8
Goodwin (2011) [53]	1	1	1	1	0	1	0	1	0	1	1	1	0	9
Meyer (2016) [22]	1	1	1	1	1	0	0	0	0	1	1	1	0	8
Plateau (2013) [54]	1	1	1	1	0	0	1	1	0	1	1	1	0	9
Plateau (2017) [55]	1	1	1	1	0	0	0	0	0	1	1	1	0	7
Sauchelli (2016) [56]	1	1	1	1	0	0	1	0	0	1	1	1	0	8
Swenne (2016) [57]	1	1	0	1	0	1	1	1	0	1	1	1	0	9
Young (2017) [58]	1	1	1	1	0	0	0	0	0	1	1	1	0	7

## Results

### Study description

The 12 studies included in this review comprise the research conducted on psychometric properties of self report measures of compulsive exercise within an eating disorder context up until 2018. Three studies focused on the Exercise and Eating Disorders (EED) [3], eight studies focused on the Compulsive Exercise Test (CET) [23], and one study included the CET and other self report measures. Of the 12 included studies, three used a non-clinical sample, six used a clinical sample as well as a non-clinical control group, and three used a clinical sample only. Diagnoses in the clinical samples included anorexia nervosa, bulimia nervosa, eating disorder not otherwise specified, binge eating disorder and other specified feeding or eating disorder. Six studies used only female participants, six studies used male and female participants, and one study focused on male participants only. Of the studies focusing on the CET, four focused on particular populations: two focused on adolescents and the other two focused on athletes. For sample characteristics and distribution of diagnoses of the individual studies as well as for relevant numeric values see Table 2.

**Table 2:** Characteristics of studies included in this review

Author (Year) (Country)	Participants	Factor structure*	Cut-off scores	Internal consistency
Studies focusing on the Exercise and Eating Disorders (EED)				
Danielsen (2012) (Norway) [3]	*N* = 50 female inpatient ED participants (57 before exclusion). AN = 25, 50%, BN = 10, 20%, EDNOS = 15, 30%, 15 participants. *N* = 51 high school and university students as control group	Items were divided into three subscales based on clinical exeperience: intentions to exercise (subscale 1), consequences of not exercising (subscale 2), bodily sensations (subscale 3). No factor analysis conducted	Cut-off score for compulsive exercise = 50, represents ≥ average score on each item given the maximal possible total of 100	Cronbach’s α in whole sample: .92 (sum score), .66 (subscale 1), .93 (subscale 2), .87 (subscale 3). Cronbach’s α in patient group: .89 (sum score), .66 (subscale 1), .94 (subscale 2), .78 (subscale 3). Cronbach’s α in control group: .79 (sum score), .39 (subscale 1), .84 (subscale 2), .80 (subscale 3)
Danielsen (2015) (Norway) [50]	*N* = 235 female ED participants (244 before exclusion), AN = 79, 32.4%, BN = 57, 23.4%, EDNOS = 84, 34.4%, BED = 24, 9.8%. *N* = 205 female controls	Four factor structure determined by PCA: (1) compulsive exercise (CE), (2) positive and healthy exercise (PHE), (3) awareness of bodily symptoms (BS) and (4) weight and shape related exercise (WSE)	Clinical severity guide: Group 1, global score < 1.80 (no symptoms of compulsive exercise); Group 2, global score 1.80–2.39 (low severity); Group 3, global score 2.40–3.19 (moderate severity) and Group 4, global score > 3.20 (high severity)	Cronbach’s α in whole sample: .90. (sum score), .93 (factor 1), .82 (factor 2), .80 (factor 3), .89 (factor 4)
Danielsen (2018) (Norway) [51]	*N* = 55 male ED in- and outpatients, AN = 30, 54.4%, BN = 10, 18.2%, and unspecified ED including binge eating = 15, 27.2%, *N* = 203 male controls included (95%) (214 before exclusion)	Four factor structure found in Danielsen (2015) was confirmed by PCA. Four factor solution explained the most variance (73%). Factor 1 (Eigenvalue 6.27) explained 34.81%, factor 2 (Eigenvalue 4.02) explained 22.33%, factor 3 (Eigenvalue 1.89) explained 10.46% and factor 4 (Eigenvalue 0.97) explained 5.39%	as in Danielsen (2015)	Conrbach’s α in whole sample: .92 (factor 1), .89 (factor 2), .88 (factor 3), .72 (factor 4)
Studies focusing on the Compulsive Exercise Test (CET)				
Goodwin et al. (2011) (United Kingdom) [53]	*N* = 1012 secondary school students 12–14 years, 1725 before exclusion, male = 45.3%, female = 54.7%	Five factor structure confirmed by exploratory PCA. Five factors explained 64.1% of variance. Factor 1 (Eigenvalue 7.89) explained 32.88%, factor 2 (Eigenvalue 2.71) 11.28%, factor 3 (Eigenvalue 1.96) 8.17%, factor 4 (Eigenvalue 1.11) 4.62% and factor 5 (Eigenvalue 1.07) 4.46%	n/a	Cronbach’s α: .88 (overall scale), .87 (factor 1), .77 (factor 2), .79 (factor 3), .71 (factor 4), .77 (factor 5)
Formby (2014) (Australia) [52]	*N* = 104 adolescents aged 12–17, 93% female, AN = 38%, BN = 11%, OFED/ USFED = 51%	Study was unable to confirm a factor structure. Four models were tested with confirmatory factor analysis, none provided adequate fit. Original five factor model suggested by Taranis et al. (2011) provided best fit out of the four	n/a	n/a
Meyer (2016) (United Kingdom) [22]	*N* = 356 female ED patients, AN = 25.9%, BN = 31% EDNOS = 38%, BED = 5%, *N* = 360 female controls	CFA for clinical sample marginally fitted 5-factor structure, however model was found to differ significantly from observed data (*Χ*^2^(242) = 768.50, *p* < .001). Removing items 8 and 12 which didn’t meet expected factor loadings didn’t improve fit. Other fit indexes marginally met criteria: RMSEA = 0.080 (90% CI = 0.073–0.086), TLI = 0.90, IFI = 0.92, CFI = 0.92	Cut-off score of 15 distinguishes between ED patients with and without features of CE	Cronbach’s α: .93 (overall scale), .96 (factor 1), .77 (factor 2), .87 (factor 3), .62 (factor 4), .82 (factor 5)
Plateau (2013) (United Kingdom) [54]	*N* = 689 competitive athletes (male = 258, female = 431). 702 before exclusion	CFA showed poor fit of five factor structure. 9 items were removed. Exploratory PCA with remaining items yielded a 3 factor solution that explained 59.90% of variance: (1) avoidance of negative affect, (2) weight control exercise and (3) mood improvement. Factor 1 explained 35.15%, factor 2 14.67% and factor 3 10.10%	n/a	Cronbach’s α: .62 (global score), .87 (avoidance of negative affect), .82 (weight control exercise), .71 (mood improvement)
Plateau (2017) (United Kingdom) [55]	*N* = 349 female athletes, *N* = 32 reported current or previous ED, *N* = 12 athletes with EDs were recruited additionally (BN = 6, AN = 3, OSFED = 3)	Factor analysis not conducted	Global CET-A score of 10 established as cut-off for identifying female athletes with an ED	n/a
Sauchelli (2016) (Spain) [56]	*N* = 157 ED participants, BN = 56, 35.7%, AN = 40, 25.5%, EDNOS = 61, 38.8%, *N* = 128 university student controls. Female = 228, 79.9%, male = 57, 20.3%	5 factor solution was supported (RMSEA = 0.087, CFI = 0.910, TLI = 0.900, SRMR = 0.080)	n/a	Cronbach’s α between .79 (ER) and .96 (ARD)
Swenne (2016) (Sweden) [57]	*N* = 254 adolescents < 18, full data available for 210, AN = 26, 12%, BN = 9, 4%, EDNOS = 175, 84%. Male = 12, female = 198	Exploratory PCA. Kaiser criterion suggested a four factor solution, scree plot3 or 4 factor solution. Final 4 factor solution explained 69.1% of variance: (1) WCE, (2) MI and (3) LEE, (4) combination of ARD and ER	n/a	Cronbach’s α: .94 (ARD), .85 (WCE), .90 (MI), .81 (LEE). Not calculated for overall scale
Taranis (2011) (United Kingdom/ Australia) [23]	3 different studies, *N* = 367 female participants, mostly university students, *N* = 101 female undergraduate students, *N* = 97 female undergraduate students respectively	Principal components analysis yielded initial 6 factor structure, changed to 5 factors after reducing to 24 items: (1) Avoidance and rule driven behavior (ARD) explained 30.39% variance, (2) Weight control exercise (WCE) 13.72% variance, (3) Mood improvement (MI), 7.71% variance, (4) Lack of exercise enjoyment (LEE), 6.74% variance, and (5) Exercise rigidity (ER), 5.32% variance	n/a	Cronbach’s α: .85 (overall scale), .88 (factor 1), .86 (factor 2), .75 (factor 3), .84 (factor 4), .73 (factor 5)
Young (2017) (Australia) [58]	*N* = 78 ED participants with AN, female = 74, male = 4	Factor analysis not conducted	n/a	Cronbach’s α: .92 (CET total), .95 (ARD), .82 (WCE), .86 (MI), .83 (LEE), .85 (ER)
Studies focusing on the Exercise and Eating Disorders (EED)				
Danielsen (2012) (Norway) [3]	Mean EED sum score patients = 58.5 (±16.5), control group 33.4. (± 11.2). Mean difference = 25.1, *p* < .001. Subscale 1 patients = 21.6 (±6.4), control group 15.8 (±4.4), mean difference = 5.9. Subscale 2 patients = 24.0 (±9.0), control group 12.1 (±6.9), mean difference = 11.9. Subscale 3 patients = 12.9 (±4.7), controls 5.6 (±3.9), mean difference 7.4. All *p* < .001		EED total (Spearman’s *ρ* = .84) and all subscales (intentions to exercise *ρ* = .65, consequences of not exercising *ρ* = .76, bodily sensations *ρ* = .71) were significantly correlated with Body Attitudes Test (BAT total) in the whole sample. All *p* < .001. Correlations between EED subscales and BAT subscales *ρ* = .55 to .76. Correlations of total scores also significant for patients and controls separately. All *p* < .001	n/a
Danielsen (2015) (Norway) [50]	EED global score patients = 2.49 (±.96), controls = 1.40 (±.65), mean difference = 1.09. Factor 1 mean score patients = 2.64(±1.40), controls 1.30 (±.95), mean difference = 1.35. Factor 2 patients = 2.41 (± 1.36), controls = 1.59 (±.1.11, mean difference = .81. Factor 3 patients = 1.86 (±1.18), controls 1.01 (±.90), mean difference = .86. Factor 4 patients = 3.00 (±1.60), controls (1.40(±.65), mean difference = 1.09. All *p* < .001		n/a	EED global score correlated significantly with EDE-Q global score for whole sample (*r* = .79) and for patients (*r* = .66) and controls (*r* = .73) separately. All *p* < .01. EED subscales CE (*r* = .70), PHE (*r* = .36), BS (*r* = .39) and WS (*r* = .65) correlated significantly with EDE-Q global score for the whole sample as well as patients and controls separately. All *p* < .01
Danielsen (2018) (Norway) [51]	EED global score patients = 2.00 (±.76), controls = 1.16 (±51), mean difference = .84. Factor 1 mean score patients = 1.89 (±1.45), controls = .65 (±.91), mean difference = 1.23. Factor 2 patients = 1.77 (±.94), controls = 1.13 (±1.08), mean difference = .64. Factor 3 patients = 2.27 (±1.40), controls = 1.56 (±1.30), mean difference = .71. Factor 4 patients = 2.16 (±1.61), controls 1.28 (±.94), mean difference = .84. All *p* < .001		n/a	EED global score correlated significantly with EDE-Q global score for whole sample (*r* = .66) and for patients (*r* = .65) and controls (*r* = .35) separately. EED CE and WSE subscales correlated significantly with EDE-Q score for the whoe sample (*r* = .65 and *r* = .61) as well as patients (*r* = .67 and *r* = .65) and controls (*r* = .39 and *r* = .54). No correlation between EDE-Q scores and PHE subscale. Only EDE-Q scores for whole male sample correlated with BS subscale (*r* = .24). *p* < .01 for all correlations
Studies focusing on the Compulsive Exercise Test (CET)				
Goodwin et al. (2011) (United Kingdom) [53]	n/a		CET total (*r* = .54,), and all subscales (ARD (*r* = .65), WCE (*r* = .27), MI (*r* = .54), LEE (*r* = −.33) and ER (*r* = .56)) were significantly correlated with CES total. All *p* < .01	EDI subscales drive for thinness (*r* = .54), bulimia (*r* = .21) and body dissatisfaction (*r* = .24) were significantly correlated with CET total. All *p* < .01
Formby (2014) (Australia) [52]	n/a		n/a	Global EDE (*r* = .68, *p* < .001) and EDI subscales body dissatisfaction (*r* = .62, *p* < .001), drive for thinness (*r* = .70, *p* < .001), bulimia (*r* = .32, *p* = .01) and perfectionism (*r* = .42, *p* = .001) were significantly correlated with CET total
Meyer (2016) (United Kingdom) [22]	CET global score patients = 14.6 (±4.71), controls = 11.4 (±3.37). Factor 1 mean score patients = 2.75 (±1.71), controls = 1.74 (±1.28). Factor 2 patients = 3.47 (±1.34), controls = 2.59 (±1.17). Factor 3 patients = 3.37 (±1.28), controls = 3.26 (±1.12). Factor 4 patients = 2.21 (±1.19), controls 1.48 (±1.09). Factor 5 patients = 2.90 (±1.55), controls = 2.37 (±1.21). All differences significant at p < .001 level except for factor 3		n/a	n/a
Plateau (2013) (United Kingdom) [54]	n/a		n/a	Strong correlations between all EDE-Q subscales and weight control exercise (*r*(685) ≥ .53), avoidance of negative affect (*r*(685) ≥ .31) and global score (*r*(685) ≥ .47). Smaller positive correlation with mood improvement (*r*(685) ≥ .16. All *p* < .01
Plateau (2017) (United Kingdom) [55]	Global CET-A score of 10 successfully discriminated female athletes with an eating disorder from those without. This cutoff score represented suitable levels of sensitivity (0.92) and specificity (0.73)		n/a	n/a
Sauchelli (2016) (Spain) [56]	Control group and all ED subgroups (AN, BN and EDNOS) differed significantly in subscales ARD, WCE and in CET total score. All *p* < .001. No differences between groups on other CET subscales		n/a	Partial correlations between CET scores and EDI. Correlations with moderate to good effect sizes are reported here. ARD, WCE and CET total correlated with drive for thinness (*r* = .408, *r =* .578 and *r* = .487), body dissatisfaction (*r =* .277, *r* = .399 and *r* = .301) and EDI total (*r* = .325, *r* = .404 and *r* = .316). ER correlated with drive for thinness (*r* = .300)
Swenne (2016) (Sweden) [57]	n/a		n/a	ARD (*R*^2^ = .27*)*, WCE (*R*^*2*^ = .53), MI (R^2^ = .08) and LEE (R^2^ = .04) all correlated with EDE-Q global score. All *p* < .001 except LEE *p* < .01
Taranis (2011) (United Kingdom/ Australia) [23]	n/a		Significant correlations (Spearman’s *ρ*) between CET total (*ρ* = .62) and subscales (ARD *ρ* = .70, WCE *ρ* = .41, MI *ρ* = .44, LEE *ρ* = −.42, ER *ρ* = .51) and Commitment to Exercise Scale (CES) total. All *p* < .001. Significant correlations between CET total (*r* = .58) and subscales (ARD *r* = .74, WCE *r* = .27, MI *r* = .36, LEE *r* = −.27 ER *r* = .52) and Obligatory Exercise Questionnaire (OEQ) total. All *p* < .01 and *p* < .001	Significant correlations between CET total, EDI total (*ρ* = .47) and EDI drive for thinness (*ρ* = .53) and body dissatisfaction (*ρ* = .40) subscales. Only the CET subscale WCE correlated with EDI total (*ρ* = .77). All *p* < .001. CET total correlated significantly with, EDE-Q total (r = .55) and EDE-Q subscales restraint (*r* = .49), eating concern (*r* = .50), shape concern (*r* = .53), weight concern (*r* = .48) All *p* < .001. CET subscales ARD (*r* = .33), WCE (*r* = .65) correlated significantly with EDE-Q total. All *p* < .001
Young (2017) (Australia) [58]	n/a		CET total correlated significantly with CES mean (*ρ* = .78), EBQ total (*ρ* = .52) and REI total (*ρ = .33).* All *p* < .01	CET total (*ρ* = .64) and CET subscales ARD (*ρ* = .72), WCE (*ρ* = .39), MI (*ρ* = .27) and ER (*ρ* = .54) correlated significantly with EDEQ total. All *p* < .01*.* No significant correlation with LEE

*Note*, *AN* Anorexia nervosa, *ARD* Avoidance and rule driven behavior, *BAT* Body Attitudes Test, *BED* Binge eating disorder, *BN* Bulimia nervosa, *BS* Awareness of bodily symptoms, *CE* Compulsive exercise, *CES* Commitment to Exercise Scale, *CET* Compulsive Exercise Test, *CFA* Confirmatory factor analysis, *CFI* Comparative Fit Index, *EDNOS* Eating disorder not otherwise specified, *ED* Eating disorder, *EDE-Q* Eating Disorder Examination Questionnaire, *EDI* Eating Disorder Inventory, *EED* Exercise and Eating Disorders, *ER* Exercise rigidity, *IFI* Incremental Fit Index, *LEE* Lack of exercise enjoyment, *MI* Mood improvement, *OEQ* Obligatory Exercise Questionnaire, *OSFED* Other unspecified feeding and eating disorder, *PCA* Principal components analysis, *PHE* Positive and healthy exercise, *REI* Reasons for Exercise Inventory, *RMSEA* Root Mean Square Error of Approximation, *SRMR* Standardized Root Mean Square Residual, *TLI* Tucker-Lewis Index, *WCE* Weight control exercise, *WSE* weight and shape exercise, *WS* Weight and shape related exercise

*Factor 1, Factor 2 etc. refers to factors identified by confirmatory or exploratory factor analysis, or principal components analysis

### Studies

#### The Exercise and Eating Disorders (EED) questionnaire

The Exercise and Eating Disorders (EED) is a self-report questionnaire designed to assess compulsive exercise as a symptom of eating disorders [3]. Three studies were found that address its development and validation. A pilot study was conducted in 2012 [3], followed by a second study in 2015 [50], which aimed to further test psychometric properties and the factor structure of the EED. A third study followed in 2018 [51], which aimed to validate the EED in a male sample. All three studies were conducted within the same research group. The EED was developed in a clinical eating disorders inpatient unit. Items and subscales were created based on clinical experience and issues typically voiced by patients in this setting. Items were initially grouped into three categories based on clinical experience without conducting factor analysis: intentions to exercise (subscale 1), which assesses reasons for exercising, consequences of not exercising (subscale 2), which assesses negative outcomes of not exercising, and bodily sensations, which assesses whether subjects notice physical sensations such as feeling hungry, thirsty or tired, and whether they take these sensations into consideration (subscale 3) [3].

#### Structure and psychometric properties

The EED is an 18 item self-report questionnaire scored on a 6-point Likert scale from 0 (*never*) to 5 (*always*). Two items were excluded after the pilot study, and three items were added in the revised version assessing quantitative aspects of exercise (frequency, intensity and duration) [50].

The EED’s factor structure, Internal consistency, test-retest-reliability, concurrent, convergent and discriminant validity and its ability to distinguish between patients and controls were tested across the three studies. Factor analysis revealed a four factor structure: Compulsive Exercise (factor 1), Positive and Healthy Exercise (factor 2), Awareness of Bodily Signals (factor 3) and Weight and Shape related Exercise (factor 4) [50]. The four factor structure was retained in the subsequent study [51]. In relation to the initial version of the EED [3], the items of the ‘consequences of not exercising’ subscale overlap with the items of the Compulsive Exercise factor on the revised version of the EED. The ‘intentions to exercise factor’ thematically overlaps with two factors on the revised version of the EED, Positive and Healthy Exercise’ and Weight and Shape Exercise. The ‘bodily sensations’ subscale has remained largely unchanged in the revised version [50]. For more detailed information about the factor structure, see Table 2.

Results from all three studies indicate excellent internal consistency for the whole scale. Internal consistency for the individual subscales ranged from acceptable to excellent except for the Compulsive Exercise subscale in the pilot study [3]. Test-retest reliability was calculated in one study. Between test and retest, Pearson’s *r* was .86 for global score and ranged between .68 and .90 for subscales. No significant differences were found between test and retest in global score and subscales [50].. Concurrent validity was assessed by correlation analysis between the EED and the Body Attitudes Test (BAT). Results show significant correlations between EED and BAT Total as well as subscales [3]. Convergent and discriminant validity were established by comparing EED and Eating Disorder Examination Questionnaire (EDE-Q) scores [50]. The EED distinguished successfully between patients and controls in all three studies. For details see Table 2.

#### The Compulsive Exercise Test (CET)

The Compulsive Exercise Test (CET) is a 24 item self-report questionnaire designed to assess exercise within an eating disorder context [23]. Nine studies were found addressing its development and validation, both within and outside of the research group in which it was initially developed. The CET’s intial development was informed by the existing literature on exercise in eating disorders, interviews with eating disorder specialists and patients and a critical appraisal of existing measures [23]. The CET adopts a multidimensional cognitive-behavioral approach, addressing concepts such as emotion regulation and compulsivity that existing research has identified to play a role in the development and maintenance of compulsive exercise [23].

#### Structure and psychometric properties

The CET is a 24-item questionnaire scored on a 6-point Likert scale ranging from 0 (*never true*) to 5 (*always true*) [23]. The CET’s factor structure, internal consistency, concurrent and convergent validity, and its ability to distinguish patients from controls were tested in the studies found.

The first version of the CET consists of 31 items and 6 factors. Seven items were excluded following initial factor analysis, revealing a five factor structure: Avoidance and Rule Driven Behaviour (factor 1), Weight Control Exercise (factor 2), Mood Improvement (factor 3), Lack of Exercise Enjoyment (factor 4) and Exercise Rigidity (factor 5) [23]. Of the nine studies that were found, two did not use factor analysis [55, 58], two studies confirmed the five factor structure [53, 56], and one study marginally confirmed the five factor structure but the model was found to be significantly different from the observed data [22]. One study was unable to confirm a factor structure at all [52], one study proposed an alternative three factor structure [54], and one study an alternative four factor structure [57]. For more detailed information about the factor structure, see Table 2.

Seven of the nine studies used Cronbach’s α to measure internal consistency [22, 23, 53, 54, 56–58]. Values ranged between questionable and excellent for the overall scale and subscales. Concurrent validity was assessed in three studies by comparing the CET to other measures of exercise behaviour, namely the Commitment to Exercise Scale (CES) [23, 53, 58], the Obligatory Exercise Questionnaire (OEQ) [23], the Reasons for Exercise Inventory (REI) [58], and the Exercise Beliefs Questionnaire (EBQ) [58]. Results show significant correlations between the measures, confirming concurrent validity of the CET. Convergent validity was assessed in seven studies [23, 52–54, 56–58] by comparing the CET to the Eating Disorder Inventory (EDI) and the Eating Disorder Examination Questionnaire (EDE-Q). Results show significant correlations between the measures, confirming convergent validity of the CET. The ability of the CET to distinguish between patients and controls was assessed in three studies [22, 55, 56]. All three found significant differences between patients and controls when considering the total score and most subscales. One study established a cut-off value of 10 to distinguish female athletes with an eating disorder from those without [55]. One study established a cut-off value of 15 [22] to distinguish between eating disorder patients with and without compulsive exercise. None of the studies on the CET included test-retest-reliability. For details see Table 2.

## Discussion

The aim of this systematic review was to examine, summarise and assess the existing research on measures of compulsive exercise which were designed to be used with eating disorder populations. Twelve studies examining two measures, the Exercise and Eating Disorders (EED) and the Compulsive Exercise Test (CET), were found. A number of psychometric parameters were investigated in these studies including construct validity, internal reliability, concurrent and convergent validity and the ability of the measures to distinguish between clinical samples and control groups.

### Strenghts

This systematic review has a number of strengths in line with the recommendations of the PRISMA checklist [48]. First, several databases were searched in order to minimise the probability of overlooking any studies that fit the criteria of this review. Second, publications in three languages were included in the search. Third, two reviewers of quality were involved in the study selection process.

The CET and the EED have also demonstrated a number of strengths throughout the studies examined in this review. The CET is the first measure of exercise designed specifically for use with eating disorder patients [23]. Given that exercise is such a prominent feature of eating disorders, a measure tailored to this population was long overdue. Furthermore, the CET is based on empirical findings from the eating disorder literature as well as on interviews with eating disorder specialists and patients and a critical appraisal of existing measures, rather than deriving its rationale from other areas such as addiction theory or substance dependence [23].. These findings indicate that weight and shape concerns [18], compulsivity [14, 19], and emotion regulation [28] play a role when eating disorder patients exercise, which is clearly reflected in the items and factor structure of the CET. The studies examined in this review have established the CET’s concurrent and convergent validity with other well-established tests such as the Commitment to Exercise Scale, Obligatory Exercise Questionnaire, Reasons for Exercise Inventory, Eating Disorder Inventory and Eating Disorder Examination Questionnaire, as well as the CET’s internal consistency. This makes the CET a promising start on the journey to establishing more specialised measures of exercise for eating disorder patients.

The EED was also designed to be used with eating disorder patients but was developed from a more practical standpoint. While both the CET and the EED took patient and clinician experiences into account, the developers of the EED did not rely on recent research or other measures of exercise to inform item development. Rather, the EED was developed by clinicians working in an inpatient eating disorder unit using a practical, patient-centered approach [3].. Items were designed based on clinical experience and on issues regarding exercise voiced by patients in the unit. Initial psychometric testing confirmed the validity of this approach [3, 50]. The EED shows good concurrent and convergent validity, and good to excellent internal reliability. The initial factor structure [50] was retained in a further study [51].

### Limitations

This systematic review has some limitations. First, grey literature was not searched, hence there may be unpublished measures that fit the search criteria applied to this review. Additionally, despite the fact that three languages were included in the search, there may be papers in other languages that were missed by the search strategy.

There were a number of limitations in the studies reviewed. First, the number of studies examining self-report measures of exercise designed to be used within an eating disorder population is small. Only 12 studies were found that met inclusion criteria. In addition, this number was not distributed evenly between the tests, with only three studies examining the EED. Results pertaining to the quality of the CET and EED should therefore be interpreted with caution. Second, sample sizes varied significantly in the included studies. Some studies had small sample sizes and did therefore not meet the recommended criteria of 10 participants per item [59] or more than 1000 participants [60] for factor analysis. Third, no power calculations were conducted in any of the studies to assess whether the number of particpants was adequate, and to avoid type II errors.

Results of the nine studies evaluating the CET in different samples such as eating disorder patients, adolescents and athletes indicate that the factor structure is somewhat unstable, and changes depending on the sample used. The initial five factor structure could not be confirmed in the majority of the studies examined in this review. The Mood Improvement subscale did not distinguish between patients and controls in three studies [22, 23, 54], potentially rendering its usefulness questionable. Despite the CET having been designed for eating disorder samples, only two of the nine studies aimed to validate the CET in an adult clinical sample. Further research and item modification may be needed to confirm a factor structure and validate the CET in more diverse clinical samples.

An issue common to both the CET and the EED is that clinical samples were mostly recruited from either inpatient facilities or other specialized eating disorder services, which puts participants at the more severe end of the eating disorder spectrum. Additionally, issues voiced by patients were taken into account when developing the EED, but may not necessarily apply to less severe presentations. It can therefore not be assumed that the tests examined in this review can be used for people with eating disorders who do not currently seek treatment, people with past eating disorders, or eating disorder patients treated in private practice.

A general limitation of the studies reviewed is that the CET and the EED have different underlying operationalisations of exercise, thereby adding to the plethora of already existing approaches and definitions. In the pilot study [3], the EED used exercising more than five times a week combined with the EED score as criteria to identify compulsive exercisers, while the CET does not include items assessing exercise frequency. While some items and subscales appear similar, there are others that do not seem to overlap. For example, both the CET and the EED include a weight and shape component, and both contain items pertaining to mood regulation. However, the EED subscale awareness of bodily signals does not appear in the CET, while the CET subscale exercise rigidity does not appear in the EED.

Additionally, while both tests appear promising, an indication of which measure is best used for different patient groups is currently lacking. The amount of research investigating the psychometric properties of the EED and CET is small. There are only three studies investigating the EED, and only two studies assessed the CET with eating disorder subjects. Given the limited amount of research into the factor structure of the EED, the inconsistent support for the factor structure of the CET, the lack of studies assessing test-retest-reliability, and the lack of studies validating both the CET and the EED in diverse eating disorder samples, it is difficult to make any recommendations for clinicians as to which measure might be best to use. Future research should aim to further validate both measures and identify which test works best for different patient groups so that such a recommendation can be made.

### Further research and outlook

Despite the progress that has been made in conceptualising compulsive exercise and identifying relevant correlates such as compulsivity and affect regulation, a widely used and accepted operational definition is still missing [4]. This may make it difficult to compare results between studies, as studies tend to differ in their definitions of compulsive exercise as well as in behaviours described to operationalise the construct. It may therefore be beneficial to conduct more research on an empirically sound working definition of compulsive exercise.

While all measures identified in this systematic review were constructed using classical test theory, there are other measurement models that might be more suitable to measuring compulsive exercise such as item response theory and generalizability theory, which would allow for separation of systematic from unsystematic errors, and for both norm-referenced and criterion-referenced outcomes [61, 62]. Future instruments may benefit from a different theoretical underpinning to explore alternative measurement models.

Additionally, while available measures define compulsive exercise as a state, it is possible to theorize that it is actually a state, or a state-trait-combination whereby the state component is subject to time or situation specific variation and is activated when an eating disorder is developed [63]. Future instruments may benefit assessing state as well as trait, allowing for a clearer conceptualisation of compulsive exercise, and adding to existing knowledge to inform assessment and treatment.

On a more practical note, recent research has indicated that carefully designed exercise interventions may be beneficial for eating disorder patients [64]. In order to be able to design and implement these interventions, it is necessary to gain understanding of the pathogenesis and maintenance factors of compulsive exercise in eating disorders. Psychometrically sound measures assessing compulsive exercise could help facilitate designing exercise interventions that are beneficial for patients’ recovery. In particular, measures could potentially be used as guidelines for recommending exercise regimes to eating disorder patients.

## Conclusion

Research has identified factors specific to exercise in eating disorders such as weight and shape concerns, affect regulation and compulsivity. Most available measures of exercise are not designed to be used specifically with eating disorder patients and therefore may not adequately measure exercise behaviours in this group. Tests specific to eating disorder patients are however essential in diagnosing and treating compulsive exercise behaviours. The CET and the EED are the only known measures of exercise designed to be used with eating disorder patients, providing researchers and clinicians with more tailored instruments. While initial validation studies showed promising results, more research is needed to further establish validity of these measures. Validating the CET and EED in more diverse eating disorder groups may help to establish them as routinely used clinical measures.

## Data Availability

The datasets analysed during the current study are available from the corresponding author on reasonable request.

## Abbreviations

AN: Anorexia nervosa
BAT: Body Attitudes Test
BED: Binge eating disorder
BN: Bulimia nervosa
CES: Commitment to Exercise Scale
CET: Compulsive Exercise Test
CFA: Confirmatory factor analysis
EBQ: Exercise Beliefs Questionnaire
ED: Eating disorder
EDE-Q: Eating Disorder Examination Questionnaire
EDI: Eating Disorder Inventory
EDNOS: Eating disorder not otherwise specified
EED: Exercise and Eating Disorders
OEQ: Obligatory Exercise Questionnaire
PCA: Principal components analysis
REI: Reasons for Exercise Inventory
